# Heat generation and light scattering of green fluorescent protein-like pigments in coral tissue

**DOI:** 10.1038/srep26599

**Published:** 2016-05-26

**Authors:** Niclas H. Lyndby, Michael Kühl, Daniel Wangpraseurt

**Affiliations:** 1 Marine Biological Section, Department of Biology, University of Copenhagen, DK-3000 Helsingør, Denmark; 2 Plant Functional Biology and Climate Change Cluster, University of Technology Sydney, New South Wales 2007, Australia

## Abstract

Green fluorescent protein (GFP)-like pigments have been proposed to have beneficial effects on coral photobiology. Here, we investigated the relationships between green fluorescence, coral heating and tissue optics for the massive coral *Dipsastraea* sp. (previously *Favia* sp.). We used microsensors to measure tissue scalar irradiance and temperature along with hyperspectral imaging and combined imaging of variable chlorophyll fluorescence and green fluorescence. Green fluorescence correlated positively with coral heating and scalar irradiance enhancement at the tissue surface. Coral tissue heating saturated for maximal levels of green fluorescence. The action spectrum of coral surface heating revealed that heating was highest under red (peaking at 680 nm) irradiance. Scalar irradiance enhancement in coral tissue was highest when illuminated with blue light, but up to 62% (for the case of highest green fluorescence) of this photon enhancement was due to green fluorescence emission. We suggest that GFP-like pigments scatter the incident radiation, which enhances light absorption and heating of the coral. However, heating saturates, because intense light scattering reduces the vertical penetration depth through the tissue eventually leading to reduced light absorption at high fluorescent pigment density. We conclude that fluorescent pigments can have a central role in modulating coral light absorption and heating.

Globally, coral reefs degrade at an alarming rate primarily because of the combined effects of climate change, pollution, overfishing and the increased frequency of coral diseases^1^,^2^,^3^,^4^,^5^. Coral bleaching describes the loss of the coral’s symbiotic algae (*Symbiodinium* spp.) and/or associated pigments and is often induced during extended periods of above average seawater temperature in combination with high irradiance^6^,^7^,^8^,^9^. Corals have different susceptibilities to bleaching, which can be related to a combination of factors such as the characteristics of the coral host, the clade of the symbiotic algae and the environmental history^10^,^11^,^12^.

Recently, it has been argued that the optical and thermal properties of corals might have a central role in defining coral photophysiology and stress susceptibility^12^,^13^,^14^,^15^,^16^,^17^. Light is scattered strongly in both coral tissue and skeleton, and such light scattering can lead to an enhancement of the *in hospite* exposure of *Symbiodinium* to photosynthetically active radiation (PAR, 400–700 nm) by about 2 times^17^. The optical properties of coral tissue and skeleton vary strongly between coral species resulting in unique light regimes for *Symbiodinium* harbored within different coral hosts^9^,^12^,^17^. Skeleton light scattering differs among species and such light scattering properties may partly explain coral bleaching susceptibility^12^. However, the optical properties of the tissue are also important, given that intense light scattering in coral tissue strongly modulates symbiont light exposure^17^. A potentially important component of radiative transfer within coral tissue are fluorescent proteins (FPs) that are synthesized by the coral host and are homologous to the well-known green fluorescent protein (GFP) from the jellyfish *Aequorea victoria*^18^.

GFP-like pigments in corals can occur in four main colors, of which three are fluorescent (cyan, green and red) and one is non-fluorescent (purple-blue)^19^. The fluorescent pigments (FPs) are synthesized by the coral in a premature and initially non-fluorescent form that can be stored in the coral tissue before maturing into the fluorescent form via an oxidation step^20^,^21^. The non-fluorescent proteins are called chromoproteins (CPs) and are characterized by minimal fluorescence and high absorption efficiency^22^,^23^. Most FPs in corals are excited by blue-green light (450–495 nm) and emit a red-shifted fluorescence (between 495–570 nm)^19^,^23^,^24^. FPs are very common in corals and it has been estimated that up to 97% of shallow water corals on the Great Barrier Reef harbor FPs^18^,^25^.

The function of FPs in corals remains unclear, albeit many suggestions have been made including photoprotection^9^,^18^, antioxidant activity^26^, and camouflage^24^. The most commonly proposed function is that FPs protect coral symbionts from excess radiation^18^,^23^. Salih *et al*.^18^ showed that corals with FPs showed higher values of the maximum quantum yield of photosystem II, i.e. the corals were significantly less photoinhibited, when exposed to high irradiance, as compared to corals without FPs. Furthermore, recent studies suggest that the expression of photoprotective FPs can be regulated depending on the degree of ambient light stress^9^. The ability to convert energy-rich solar radiation, especially damaging ultra violet (UV) radiation, into red-shifted less harmful radiation has thus been suggested to be a key aspect of the photoprotective characteristics of FPs^18^. Although photoprotection is apparently an important function of FPs in light stressed corals, they might at the same time stimulate photosynthesis in low light habitats^27^,^28^,^29^, and corals located in deeper waters were found to harbor FPs primarily in lower tissue layers^27^. It has been speculated that light transmitted through the tissue can be backscattered by FPs located deeper within the tissue, thus potentially enhancing photon absorption by the symbionts located above FPs^30^, but such a function of FPs in corals still awaits experimental confirmation.

While GFP-like pigments can affect the optical properties of corals in various ways, it has so far not been considered, whether FPs can also affect the thermal properties of corals. The temperature microenvironment is directly linked to the light absorption properties of an organism. When light is absorbed it can either result in a chemical reaction (e.g. photosynthesis), be re-emitted as light (via scattering or fluorescence) or be converted into heat (i.e. random molecular vibrations). The total temperature rise (or warming) of an organism is directly proportional to the total radiative energy absorbed^31^. In corals, about 90% of the absorbed light energy is converted to heat^32^. The application of temperature microsensors has facilitated an understanding of the local heat generation of photosynthetic underwater organisms such as corals and biofilms^14^,^33^. Such measurements have shown that the coral surface temperature can increase by up to 1 °C relative to the ambient water mass^14^. The specific coral tissue surface temperature is a function of heat generation, i.e., light absorption, as well as heat transfer to the coral skeleton and to the overlaying water through the thermal boundary layer^14^,^34^. Heat generation has been demonstrated to be primarily a function of *Symbiodinium* light absorption in corals with low FP content, e.g., the brown morphs of *Porites lobata* and *Stylophora Pistillata*^15^. The thermal action spectrum of coral heating showed that heating correlated with the peak absorption of Chl *a* (between 400–450 nm and 650–680 nm)^15^.

Coral FPs have been suggested to interfere with *Symbiodinium* light absorption through various optical mechanisms including scattering, fluorescence and light absorption^18^,^35^,^36^,^37^. For instance, some FPs absorb blue light within the peak absorption of *Symbiodinium* (~420 nm) and re-emit part of this light as green fluorescence. Compared to blue light, absorption of green light is lower for *Symbiodinium*, and green fluorescence could thus interfere with the coral heat budget. Additionally, intense reflection of blue-green light by FPs might lower heating by reducing coral light absorption. However, some FPs have high molecular extinction coefficients^19^ and could also lead to enhanced heating of corals. So far the relationship between FP content and coral heating has not been explored, albeit such knowledge could provide important insight into the regulation of the optical and thermal microenvironment and radiative energy budget of corals and thereby coral bleaching susceptibility.

In this study, we used a combination of fiber-optic and electrochemical microsensors along with imaging of variable chlorophyll fluorescence, green fluorescence and hyperspectral imaging to investigate relationships between green fluorescence, coral heating and coral microscale optics. Specifically, we investigated i) the tissue surface distribution of green fluorescence at high spatial resolution, ii) the potential relationships between green fluorescence, coral heating and tissue scalar irradiance, and iii) the wavelength dependency of heating and scalar irradiance enhancement in a massive thick-tissued coral.

## Methods

### Corals

Sun-adapted corals were collected from shallow waters (<2 m depth) on the reef flat of Heron Island, Great Barrier Reef, Australia (152°06′E, 20°29′S). On a cloudless day during mid-day sun, the incident downwelling photon irradiance reaches ~2000 μmol photons m^−2^ s^−1^ and is composed of a similar contribution of light between 400–700 nm^38^. A colony of the coral *Dipsastraea sp*. (previously *Favia sp*.) was selected as this species is known to harbor FPs^19^,^28^. The colony was transported to the coral holding facility at the University of Technology, Sydney, where it was maintained under continuous flow of seawater at 25 °C and a salinity of 35.

### FP upregulation

In order to provide a wide range of measurement locations, i. e., tissue areas with different FP content, we stimulated FP upregulation through enriched blue light treatment. D’Angelo *et al*.^39^ showed that when corals were grown with the same photon irradiance (200 μmol photons m^−2^ s^−1^) of red, green or blue light, a 2-fold higher density of GFP-like proteins was found in corals incubated with blue light. The *Dipsastraea* sp. colony was split in two using hammer and chisel, where after each fragment was cultured for about 6 weeks under one of two spectral regimes: i) white light plus blue light to induce tissues areas with greater levels of green fluorescence (HF-‘highly fluorescent’), and ii) white light only to culture tissue areas with moderate green fluorescence (NF-‘normal fluorescent’). We used the same colony in order to reduce the chance that factors related to biological variability such as symbiont genotype and coral growth history would confound the relationships between green fluorescence and coral heating/optics. A culture period of about 6 weeks was chosen to ensure sufficient upregulation of FP synthesis. The incident downwelling photon irradiance was adjusted to 200 μmol photons m^−2^ s^−1^ (400–700 nm; 12/12 h light-dark cycle) for both spectral regimes (as in D’Angelo *et al*.^39^) and was measured by a light meter (LI-189, Li-COR, USA) equipped with a quantum sensor (LI-190R, Li-COR, USA). We chose the same photon irradiance in order to minimize the chance that differences in light intensity induce changes in *Symbiodinium* density.

### Experimental setup and approach

For all experiments, coral fragments were placed in an acrylic flow chamber that was connected to a 30 L reservoir tank via a submersible water pump that supplied seawater (25 °C, salinity = 35) at a flow velocity of ~0.5 cm s^−1^. A motorized micromanipulator (MU-1, PyroScience GmbH, Germany) was attached to a heavy-duty stand to facilitate positioning of scalar irradiance and temperature microsensors on the coral surface (see below for details). Placement of the microsensor tips onto the coral tissue surface was done visually with the aid of a dissecting microscope. Illumination was provided vertically from above by either a programmable spectral light engine or a tungsten halogen lamp as described below.

**Figure 1:**
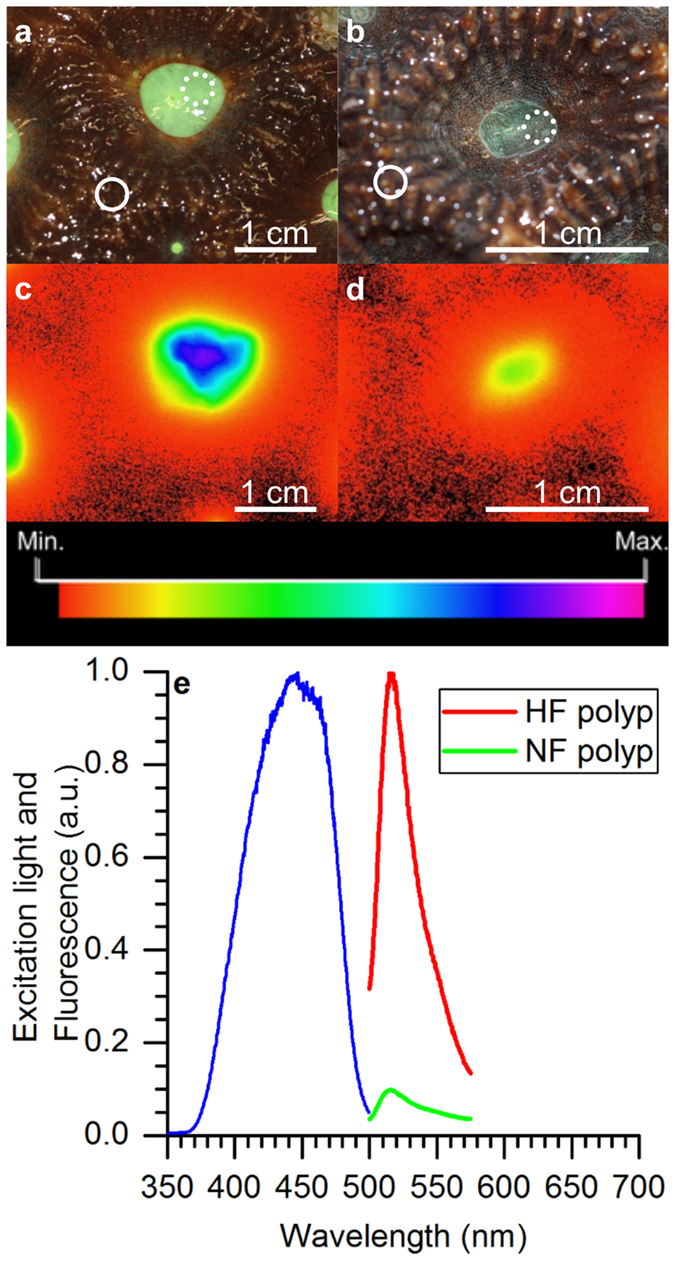
Green fluorescence emission of *Dipsastraea* sp. Representative images of (**a**,**c**) the highly fluorescent (HF) fragment, and (**b**,**d**) the normal fluorescent (NF) fragment. (**a**,**b**) White light illumination images of typical polyp (dotted circles) and coenosarc (solid circles) measurement locations. Measurements over coenosarc tissue were performed at the intersection of three neighbouring polyps and measurements on polyp tissues avoided the immediate polyp mouth opening (see methods). (**c**,**d**) Images of green fluorescence (500–575 nm) emission following blue light excitation (peaking at 480 nm). The aperture was f/2 and f/1.4 for (**c**,**d**) respectively. (**e**) Spectral characteristics of Dipsastraea sp. showing the spectrum of the broadband excitation source (blue line; peak = 450 nm) and emission spectrum (peak = 516 nm) for HF polyp (red) and NF polyp (green). White scale bars indicate 1 cm.

Microsensor measurements of scalar irradiance and temperature as well as imaging of green fluorescence and variable chlorophyll *a* fluorescence were performed on three randomly chosen coenosarc (tissue connecting polyps) and polyp tissue areas of each fragment, i.e., a total of 12 measurement locations. In order to standardize our measurement locations and account for changes in microscale topography, coenosarc measuring spots were chosen at the intersection between three polyps, and polyp measuring spots avoided the vicinity of the mouth opening (see Fig. 1a,b for measurement locations). Each chosen measurement location was identified through imaging allowing us to correlate green fluorescence to tissue heating and scalar irradiance for the exact same measurement areas. We thus aimed to reduce the effects of microscale heterogeneity through repeated fine scale measurements on identical tissue areas.

### FP imaging

We used green fluorescence (500–575 nm) imaging as indicator of relative FP content in coral tissues^18^,^40^. Previous studies have shown that there is a strong positive correlation between green fluorescence intensity and FP concentration in coral tissues^37^,^39^,^41^. While the contribution to green fluorescence between 500–575 nm in corals is dominated by GFP-like pigments, there are some GFP-like pigments that do not emit green fluorescence such as chromoproteins as well as the premature stage of some GFP-like pigments^37^. In our study, the use of green fluorescence as a proxy for relative FP density is thus limited to the fluorescent forms (FPs) of GFP-like pigments. Additionally, blue light absorbed by zooxanthellae before it reaches gastrodermal FPs is likely to reduce the intensity of the imaged green fluorescence^37^,^42^. Thus the imaging approach will more closely relate to the surface density of FPs. However, our imaging technique allows rapid, noninvasive assessment of green fluorescence intensity for a given tissue area of interest and enables close alignment of such imaging to the local thermal and optical microenvironment as measured with microsensors.

We imaged green fluorescence from coral surfaces using a pulse-amplitude-modulated (PAM) chlorophyll fluorescence imaging system (Imaging-PAM, blue LED/GFP Mini I-PAM, Heinz Walz GmbH, Germany). The I-PAM was equipped with a bandpass filter that had a 500–575 nm transmission range (K6-MIN/FS, Walz GmbH, Germany) in order to capture green fluorescence emitted by GFP-like proteins following blue light excitation (λ_max_ 470 nm). The imaging system was attached to a heavy-duty stand and mounted vertically above the flow chamber with a working distance of 5 cm between the camera lens and the coral surface. The camera aperture was set to f/1.4 and the manual focus was fixed. FP imaging was performed by darkening the coral fragment in the flow chamber for 5 min before application of a brief saturation pulse (2500–3000 μmol photons m^−2^ s^−1^ for 0.8 s) to capture a single image. No other light was applied apart from the saturation pulse.

In a few cases, the fluorescence signal was oversaturated under the chosen aperture settings (f*/*1.4). As the amount of light detected by the imaging system is directly proportional to the aperture area, the measurements were re-done with a smaller aperture area by increasing the f-number from f/1.4 to f/2–8. To allow comparison of the FP signal obtained for different f-numbers the image values had to be calibrated. This was done by imaging a given measurement spot for all f-numbers (i.e. f/2, f/2.8, f/4, and f/8) and calculating the aperture area as:


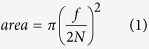


where *f* is the focal length (16 mm) and N is the f-number, i.e., the focal length divided by aperture diameter. The aperture area, i.e., the area of the coral observed by the I-PAM camera, was fitted against the image value, using a linear fit (R^2^ > 0.99) to calculate the image value for f/1.4. Fluorescence data was normalized to the maximum measured fluorescence.

### Excitation-emission spectra and hyperspectral imaging

Broadband excitation-emission spectra of one polyp of both HF and NF fragment were collected using a field radiance probe^43^ (diam. 1.5 mm) connected to a spectrometer (USB2000+, Ocean Optics, USA). Incident blue light was provided by a fiber-optic tungsten-halogen lamp (KL 2500, Schott GmbH, Germany) fitted with a blue bandpass filter (λ_max_ 460 nm). The field radiance probe was positioned 5 mm from the polyp tissue surface, at an angle of 45° relative to the vertically incident light.

The HF and NF *Dipsastraea* sp. fragments were imaged under a dissection microscope (SZ X16, Olympus, Japan), equipped with a hyperspectral image scan unit (VNIR-100, Themis Vision Systems, USA)^44^, in order to obtain high-resolution spectral images of the coral tissue surface. A single polyp and coenosarc area of both the HF and NF fragment were imaged for hyperspectral reflectance using white light illumination (using the KL 2500, Schott GmbH, Germany) and hyperspectral fluorescence (same light source equipped with a blue bandpass filter as above). Hyperspectral images were analysed using the manufacturer’s software HyperVisual 3.0 (Themis Vision Systems, USA).

### Variable Chl *a* fluorescence imaging

Variable Chl *a* fluorescence imaging was performed using the I-PAM in the standard configuration, i.e., without the transmission filter. The blue LED’s (λ_max_ 470 nm) of the imaging system provided defined actinic light levels of incident downwelling photon irradiance (PAR, 400–700 nm) of 0, 4, 29, 84, 176, 347, 547, and 692 μmol photons m^−2^ s^−1^ as calibrated by measurements of downwelling irradiance with a light meter (LI-189, Li-COR, USA) equipped with a spherical quantum sensor (US-SQS/L, Heinz Walz GmbH, Germany). For each measurement, the sample was dark acclimated for at least 5 minutes before a saturating pulse (2500–3000 μmol photons m^−2^ s^−1^ for 0.8 s) was applied to determine the maximum quantum yield of PSII. Thereafter, the samples were incubated at 5 min intervals for each experimental photon irradiance level before a saturation pulse was applied at the end of each incubation period. After measurements under actinic irradiance, the coral fragment was darkened for 5 minutes (followed by a saturation pulse) and darkened for another 5 minutes (followed by a final saturation pulse); this was done to investigate the recovery of the photosystem following high light exposure. The effective quantum yield of photosystem II [Y(II)], the quantum yield of regulated non-photochemical energy loss in PSII [Y(NPQ)] and the quantum yield of non-regulated non-photochemical energy loss in PSII [Y(NO)] were calculated as described previously^45^.

### Wavelength-dependent tissue surface warming

Coral heating was measured using two temperature microsensors (tip diameter = 50 μm, Unisense A/S, Denmark)^14^ simultaneously. Both sensors were connected to a thermocouple meter (Unisense A/S, Denmark). One microsensor was mounted on the micromanipulator oriented at a 15° angle relative to the vertical incident light beam to measure the temperature at the coral surface. The other sensor measured the temperature in the ambient water near the outlet of the flow chamber. The second temperature microsensor thus acted as a reference sensor to account for potential changes in the ambient temperature and signal fluctuations related to electrical noise. The action spectrum of coral heating was performed at an ambient water temperature of 25 °C. Microsensors were linearly calibrated against a thermocouple meter fitted with a temperature wire probe (FLUKE 52ii, FLUKE Inc., USA) from readings in seawater kept at 15 °C and 25 °C. For coral measurements, the temperature microsensor tip was carefully positioned at the tissue surface using the micromanipulator.

A programmable spectral light engine (OL-490 Agile Light Source, Gooch & Housego, USA)^46^ was used to provide spectrally well-defined illumination of known irradiance. The OL-490 delivered 11 different spectra in the wavelength range of 425–675, using increments of 25 nm, and a fixed bandwidth of 50 nm. The total photon irradiance output for each of the 11 spectra was 418 μmol photons m^−2^ s^−1^ (±1 μmol photons m^−2^ s^−1^) as measured with a calibrated spectroradiometer (JAZ-ULM, Ocean Optics, USA). The spectroradiometer also measured the output power, which allowed for expressing coral heating either per incident downwelling photon irradiance (in μmol photons m^−2^ s^−1^) or per incident downwelling irradiance (in W m^−2^).

The action spectrum of heating was measured by quantifying the increase in coral tissue surface temperature upon spectral illumination relative to the surface temperature in darkness. Illumination for each spectral range was provided for 5 min, starting with the lowest wavelength range, i. e., 400–450 nm (peak at 425 nm). To avoid that light pre-exposure affected heating, the samples were kept for 5 minutes in darkness in-between each spectral treatment. During the experiment, a calibrated temperature minisensor connected to a data logger (Firesting, PyroScience GmbH, Germany) was attached near the inlet of the flow chamber, to log the temperature of the ambient water continuously every 5 seconds. Tissue surface heating was expressed as ΔT, i. e., the difference between coral surface temperature and the temperature in the ambient water, for each spectral band and normalized for equal irradiance or equal photon irradiance^15^,^47^.

### Wavelength-dependent tissue surface scalar irradiance enhancement

To relate the wavelength-dependent thermal response of corals to their optical properties, we also characterized the tissue surface light field with scalar irradiance microprobes^48^. The microprobes were mounted on a micromanipulator oriented at a 45° angle relative to the light beam in order to avoid self-shading. The OL-490 light source was used to illuminate the coral fragments at 580 μmol photons m^−2^ s^−1^ (±5 μmol photons m^−2^ s^−1^) for each of 14 different light spectra, ranging from 420–680 nm (in increments of 20 nm with a bandwidth of 30–38 nm). The incident downwelling irradiance was measured for each measurement spot under identical conditions of illumination over a black light absorbing surface (Fig. S1e)^16^. The scalar irradiance emitted by the OL-490 was calibrated against a light meter (LI-189, Li-COR, USA) equipped with a spherical quantum sensor (US-SQS/L, Heinz Walz GmbH, Germany). The spectral scalar irradiance was recorded with the microprobes connected to a PC-connected fiber-optic spectrometer controlled by the manufacturer’s software (USB 2000+ and SpectraSuite, Ocean Optics, USA).

The enhancement of tissue surface scalar irradiance (E_o_) relative to the incident downwelling irradiance (E_d_) was calculated for each excitation spectrum, i. e., between 420–680 nm. Scalar irradiance enhancement factors were calculated by integrating the mathematical area in the scalar irradiance spectra (between 400–700 nm) measured on the coral tissue surface (E_0_) and dividing it by the respective integral value obtained from the E_d_ spectra. Because the excitation spectra followed a Gaussian distribution (R^2^ > 0.99) over a narrow waveband (Fig. S1e), the fluorescence contribution to E_o_ enhancement was approximated based on the Stokes shift relative to the Gaussian (Fig. S2). Data points indicative of fluorescence between 420–620 nm were removed and the spectra were fitted to a Gaussian distribution based on a non-linear least squares Levenberg–Marquardt iteration algorithm (R^2^ > 0.98; see example in Fig. S2). The fluorescence contribution was then expressed as percentage of the E_o_ enhancement factor. Our estimates are conservative and probably represent underestimates, as any small fluorescence contribution that would overlap with the peak excitation of the given waveband would not be captured. However, given the narrow wavebands used, such a contribution would be small.

### Statistical analyses

Simple correlation analyses were used to test for relationship between FP fluorescence, coral heating and PAR enhancement. One-way analysis of variance (ANOVA) was used to test for statistical differences in wavelength dependent heating. Assumptions for homogeneity of variance and normality were tested using Cochran’s C test and Shapiro-Wilk test, respectively. Student’s t-test was used to test for differences in variable chlorophyll *a* fluorescence parameters [i.e. Y(ii), Y(NPQ), Y(NO)] between different tissue types. Statistical tests were performed in *Statistica* V 10 (Statsoft, USA).

## Results

### FP imaging

Incubation of *Dipsastraea* sp. under ‘white plus blue light’ (HF fragment) stimulated green fluorescence between 500–575 nm (Fig. 1). Compared to the white light incubated NF fragment, green fluorescence of the HF fragment was on average 10 and 2.5 times higher for polyp and coenosarc tissues, respectively (Table 1 and Fig. 1c–e). Broadband excitation with blue-green light (350–500 nm, peaking at 440 nm) resulted in emission of green fluorescence peaking at 520 nm (Fig. 1e).

### Hyperspectral imaging

**Figure 2:**
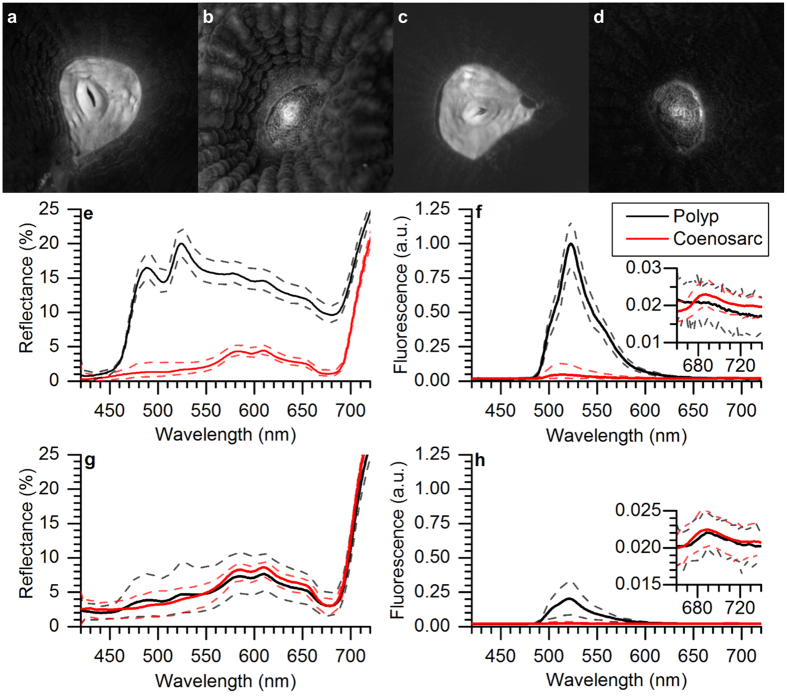
Hyperspectral imaging of reflectance and fluorescence of *Dipsastraea* sp. (**a**–**d**) Hyperspectral images showing reflectance at 520 nm (**a**,**b**) and fluorescence (about 520 nm) (**c**,**d**) of a single polyp and surrounding coenosarc tissue area for a HF fragment (**a**,**c**) and a NF fragment (**b**,**d**). (**e**,**f**) Reflectance and fluorescence spectra of a HF fragment and (**g**,**h**) reflectance and fluorescence spectra of a NF fragment (blue excitation λ_max_ ~ 460 nm) extracted from hyperspectral image stacks for polyp and coenosarc tissue areas of interest. Inserts in (**f**,**h**) shows the much weaker Chl *a* fluorescence within the same investigated areas. Solid lines represent means and dashed lines the standard error of the mean (n = 3).

Hyperspectral fluorescence and reflectance imaging showed that the FP was distributed in granula, i. e., aggregations of pigments, with highest density in the polyp regions and more sparsely granulated regions over the coenosarc tissues (white spots in Fig. 2a–d). The hyperspectral reflectance of visible light for the HF fragment was about 5.5 times higher for polyp vs. coenosarc tissues, while there was no difference between tissue types (i.e. between polyp and coenosarc) for the NF fragment (Fig. 2e,g). Reflectance imaging of the polyp tissue of the HF fragment showed spectral peaks at 475 nm and 520 nm, indicative of cyan and green FPs, respectively (Fig. 2e). For the polyp tissues of the HF fragment, fluorescence was about 75% higher than the respective tissues of the NF fragment (Fig. 2f,h). The contribution of green fluorescence from coenosarc tissue areas was small (0.13% and 0.04%, HF and NF respectively, Fig. 2f,h). Chlorophyll *a* fluorescence was detectable in all scans but was less than 0.03% of the peak fluorescence (see inserts in Fig. 2f,h).

### Variable Chl *a* fluorescence

**Figure 3:**
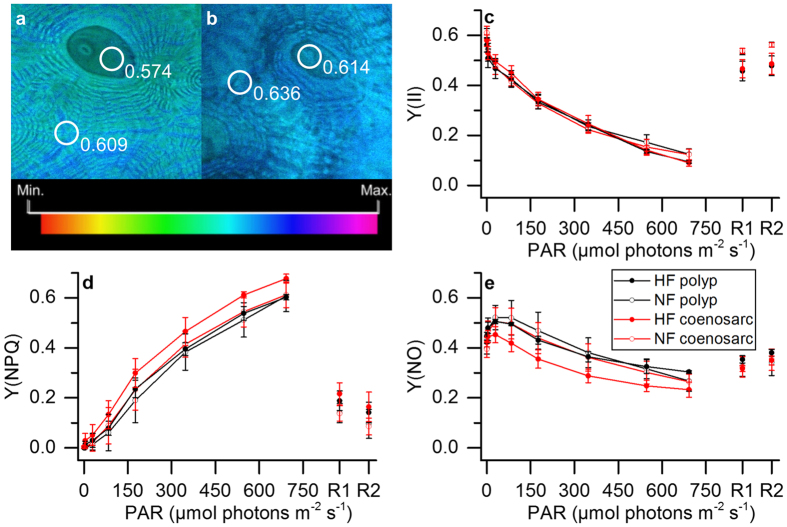
Variable chlorophyll fluorescence imaging of *Dipsastraea* sp. (**a**,**b**) Images of the maximum quantum yield of PSII, for the HF (**a**) and the NF fragment (**b**). Images were color coded to the same dimensionless scale. (**c**–**e**) Steady state light curves of Y(II), Y(NPQ) and Y(NO) ((**c**–**e**) respectively). Symbols and error bars represent mean values ± s.e.m. (*n* = 3). Recovery pulses were applied after 5 (R1) and 10 min (R2) of darkening (*n* = 3).

The maximum PSII quantum yield was similar for the different tissues and ranged on average between 0.57–0.61 (Fig. 3a,b). Likewise, no differences were found in the effective quantum yield of PSII between tissues, despite different levels of green fluorescence (Fig. 3b). However, the pathways of regulated (NPQ) and non-regulated (NO) energy dissipation revealed minor but significant differences between HF polyp and HF coenosarc tissues under high levels of incident irradiance (Fig. 3d,e). Compared to HF coenosarc tissues, the HF polyp tissues showed about 8% lower NPQ (t-test t = −6.3, DF = 4, p = 0.003) and 7% higher NO (t-test t = 3.95, DF = 4, p = 0.02) at high incident photon irradiance levels (700 μmol photons m^−2^ s^−1^, Fig. 3d,e). There was no difference in NPQ at high irradiance between HF polyp tissues and the other tissue types.

### Coral surface heating

**Figure 4:**
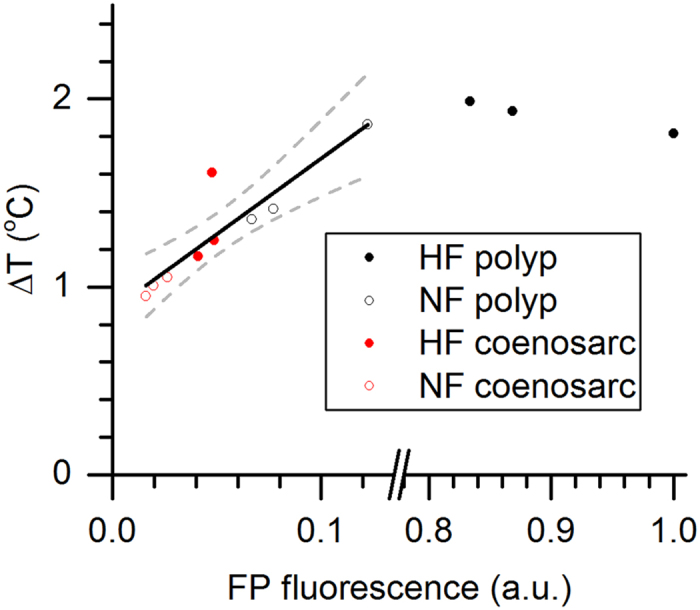
Relationship between green fluorescence and coral tissue surface heating. Coral tissue surface heating is expressed as ΔT (°C), i.e., the difference between coral surface temperature and the temperature in the ambient water, and was integrated for excitation between 400–700 nm (PAR) and expressed for a total irradiance delivery of 500 W m^−2^. FP fluorescence (a.u.) was normalized to the maximally measured fluorescence (=1). The black line shows the positive correlation between heating and green fluorescence (R^2^ = 0.89). Dashed lines are 95% confidence intervals.

**Figure 5:**
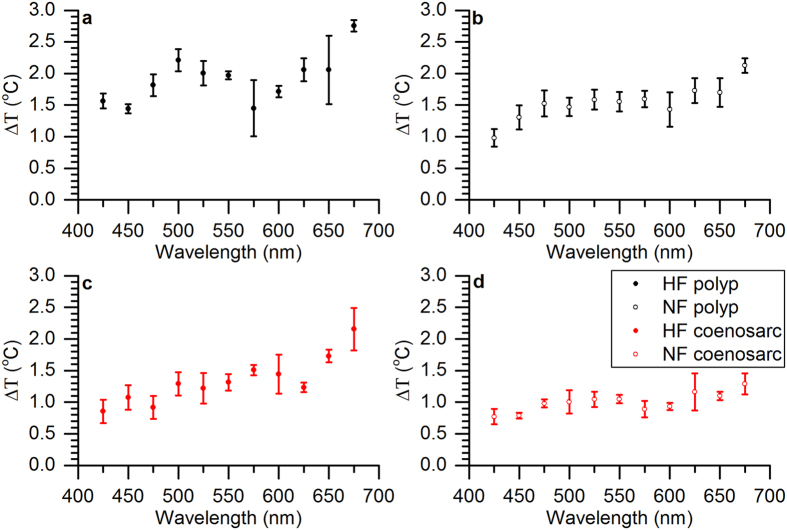
Coral thermal action spectrum. Tissue surface heating is expressed as ΔT (°C), i.e., the difference between coral surface temperature and the temperature in the ambient water, and was normalized for an equal irradiance of 500 W m^−2^ over each spectral band. Measurements were performed for (**a**) HF polyp, (**b**) NF polyp, (**c**) HF coenosarc and (**d**) NF coenosarc tissues (*n* = 3).

We found a positive correlation (R^2^ = 0.90) between coral surface heating and green fluorescence for relative green fluorescence levels ranging between 0–0.2, while heating saturated at higher levels of green fluorescence (Fig. 4). The thermal action spectrum of coral surface heating vs. spectral irradiance (in W m^−2^) revealed that on average heating decreased with shorter wavelengths; heating was highest in the red, intermediate in the green and lowest in the blue (Fig. 5). We specifically compared differences in heating for excitation spectra overlapping with the Chl *a* peak at 425 nm and 675 nm. Except for the case of lowest green fluorescence in the NF coenosarc tissues, heating was significantly higher in the red light region (one-way ANOVA, p < 0.05, Figs 5 and S3). For instance, for the HF polyp tissue, mean heating at 675 nm (2.76 ± 0.09 S.E.M.) was about 1.7 times higher compared to heating at 425 nm (1.57 ± 0.12 S.E.M. one-way ANOVA, p < 0.01). For the NF coenosarc, tissue surface heating was on average 1.2 times higher but this difference was not statistically significant (one-way ANOVA, p = 0.06).

### Coral tissue surface scalar irradiance enhancement

**Figure 6:**
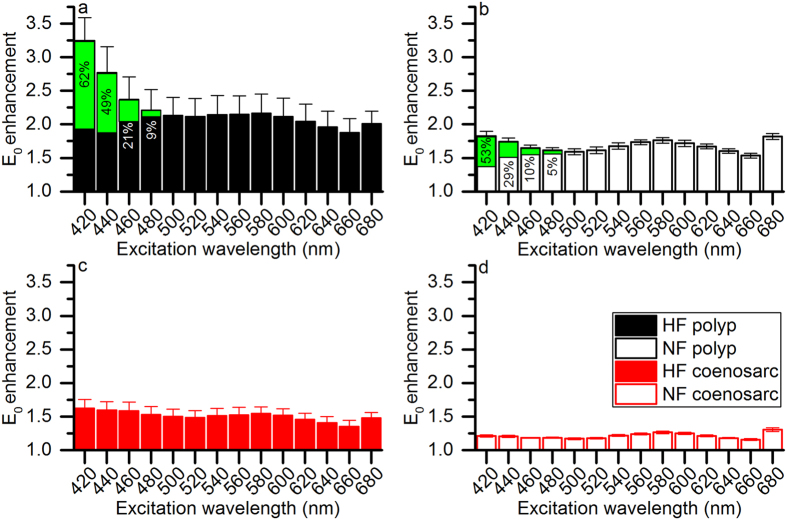
Coral tissue surface scalar irradiance enhancement as a function of excitation spectra. Scalar irradiance enhancement factors were calculated by integrating the mathematical area (between 400–700 nm) under the scalar irradiance raw spectra measured on the coral tissue surface (E_0_) and dividing it by the respective value extracted from the E_d_ spectrum. The contribution of green fluorescence to scalar irradiance enhancement is shown in green area and indicated as % of total enhancement for each spectral band. Note that minor green fluorescence contributions of <5% are not shown. Measurements were performed for (**a**) HF polyp, (**b**) NF polyp, (**c**) HF coenosarc and (**d**) NF coenosarc tissues (*n* = 3).

Photon scalar irradiance at the coral tissue surface was enhanced over the incident downwelling irradiance for all investigated measurement spots and different spectral wavebands of incident light (Figs 6a–d and S1). We found a wavelength-dependency of E_0_ enhancement, which was dependent on the different investigated tissues (Fig. 6a–d). For the HF polyp tissue, E_0_ enhancement was maximal when excited with 420 nm light (E_0_ enhancement = 3.24 times ± 0.35 S.E.M.) and E_0_ enhancement gradually decreased when excited with longer wavelength light (Fig. 6a). In contrast, maximal E_0_ enhancement for the NF coenosarc tissue was found when excited with 680 nm light (E_0_ enhancement = 1.31 times ± 0.03 S.E.M., Fig. 6d). For the HF and NF coenosarc tissues, the contribution of blue-green and green fluorescence to E_0_ enhancement was <5% for all excitation spectra. In contrast, for the HF and NF polyp tissues blue-green and green fluorescence contributed maximally at 420 nm excitation with up to 62% and 49% of the total E_0_ enhancement, respectively (Fig. 6a,b).

### Comparison of scalar irradiance enhancement and tissue surface heating

**Figure 7:**
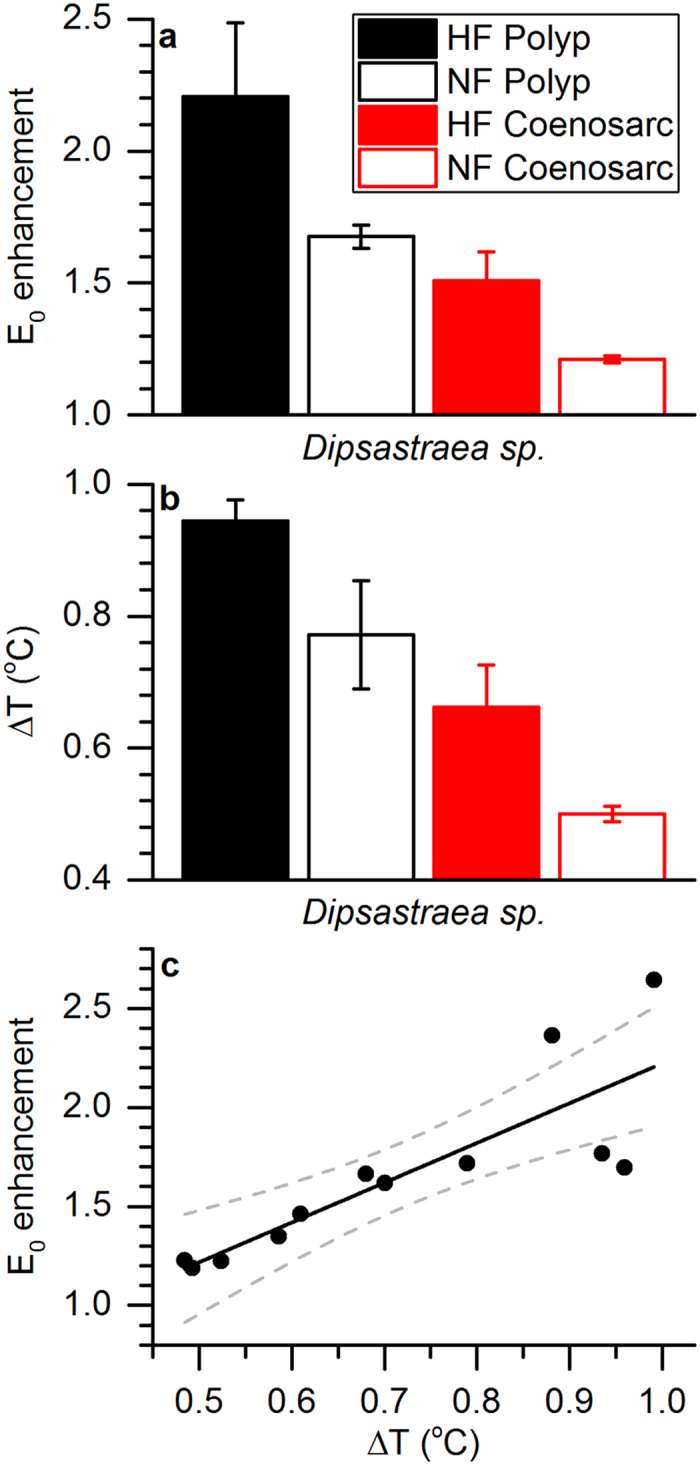
Coral tissue surface scalar irradiance enhancement and tissue heating. (**a**,**b**) Average values for (**a**) coral tissue surface scalar irradiance (E_0_) enhancement factors and (**b**) tissue heating (ΔT, °C) both for excitation integrated between 400–700 nm. Columns are arranged in order of descending measured green fluorescence (i.e. HF polyp, NF polyp, HF coenosarc and NF coenosarc) and represent means ± s.e.m. (*n* = 3). (**c**) Positive correlation between tissue surface scalar irradiance enhancement and tissue heating. The black line shows a linear fit and the dotted lines represent the 95% confidence intervals (R^2^ = 0.84).

We integrated the E_0_ enhancement and tissue heating over PAR (400–700 nm) and compared the average response of the different tissues. We found a clear hierarchy depending on the green fluorescence levels of the tissues, and both tissue heating and E_0_ enhancement showed identical patterns. Maximum and minimum E_0_ enhancement was found for the HF polyp tissue (2.21 times ± 0.28 S.E.M.) and the NF coenosarc tissue (1.21 times ± 0.01 S.E.M.), respectively (Fig. 7a). Likewise, tissue surface heating was maximal and minimal for the HF polyp tissue (0.94 ± 0.03 S.E.M.) and the NF coenosarc tissue (0.50 ± 0.01 S.E.M.), respectively (Fig. 7b). Values for NF polyp and HF coenosarc were intermediate, with an E_0_ enhancement factor of 1.68 times ± 0.04 S.E.M. and 1.51 times ± 0.11 S.E.M. and a surface heating of 0.77 ± 0.08 S.E.M. and 0.66 ± 0.06 S.E.M., respectively. There was a positive correlation between tissue surface PAR enhancement and tissue surface heating (Fig. 7c, R^2^ = 0.84).

## Discussion

The function of fluorescent pigments in coral photobiology is unclear and previous studies suggested various functional roles of FPs including photoprotection, photosynthesis-stimulation and antioxidant activity (see^49^ for review). A key knowledge gap in understanding the function of FPs relates to their potential role in modulating the optical and/or thermal properties of corals. Here we present the first experimental data linking green fluorescence to the thermal and optical microenvironment in coral tissue. We show that both temperature and tissue scalar irradiance (400–700 nm) are enhanced in tissues with higher green fluorescence. Heating saturates at high values of green fluorescence and these observations are in the following explained according to light scattering theory in biological tissues.

Surface scalar irradiance (400–700 nm) was enhanced in coral tissues with higher levels of green fluorescence (Figs 6a–d and 7). Although the putative role of FPs for enhancing PAR has long been suggested^30^, combined measurements of tissue scalar irradiance and green fluorescence have so far been lacking. Previous studies determined only reflectivity^18^ and/or fluorescence (e.g.^27^). For instance, Salih *et al*.^18^ showed that tissue reflectivity increased with FP content supporting the notion of strong light scattering by FPs. The data presented here strongly support these earlier observations and demonstrate that coral FPs can scatter light strongly.

Microscale scalar irradiance measurements showed significant enhancements of coral tissue surface PAR in relation to green fluorescence for all spectral bands of incident light (Fig. 6a–d). Light scattering occurs because of fluctuations in the refractive index within tissues and is ideally wavelength independent when the scattering particles are in the order of the wavelength of visible light^50^. In corals, cyan and green FPs often occur in granules or chromatophores with a size category in the order of wavelengths in the PAR region, i.e., about 0.5–1 μm in diameter^18^,^27^,^51^. Given the high density of fluorescent protein that occurs in the granules^51^,^52^, the refractive index of such dense aggregates of FPs is likely to be higher than the refractive index of the surrounding matrix and cells. FPs in such a granule configuration would represent good so-called Mie scatterers exhibiting wavelength independent scattering^50^, which would explain the observed enhancement of tissue surface PAR over all spectral bands of excitation light (Fig. 6a–d).

We found a positive relationship between tissue PAR enhancement and tissue heating (R^2^ = 0.84, Fig. 7c). When radiative energy interacts with a biological tissue, part of the absorbed light energy is dissipated as heat, and the local heat generation is primarily a function of incident light energy and the light absorption properties of the tissue. Mathematically, heat generation is defined as: (2) 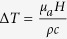, where *μ*_*a*_ is the absorption coefficient (cm^−1^), *H* is the radiant energy exposure (J cm^−2^), ρ is the density (g cm^−3^), and c is the heat capacity (J °C^−1^ g^−1^) of the tissue^31^,^47^. However, in strongly light scattering tissues, such as coral tissues, the local scalar irradiance can exceed the incident radiation by several times^16^,^53^. In such cases, heating is a function of the local scalar irradiance (E_0_) and not the incident radiative exposure (H)^31^. This would thus suggest that coral tissues with enhanced green fluorescence and thus enhanced surface PAR would also generate more heat. Indeed, we found a significant positive correlation (R^2^ = 0.90) between green fluorescence and tissue heating at low to intermediate levels of green fluorescence (Fig. 4). Thus as the density of light scattering fluorescent pigment granules increases, so does the local tissue scalar irradiance, which subsequently enhances the chance of light absorption and heating by the coral.

**Figure 8:**
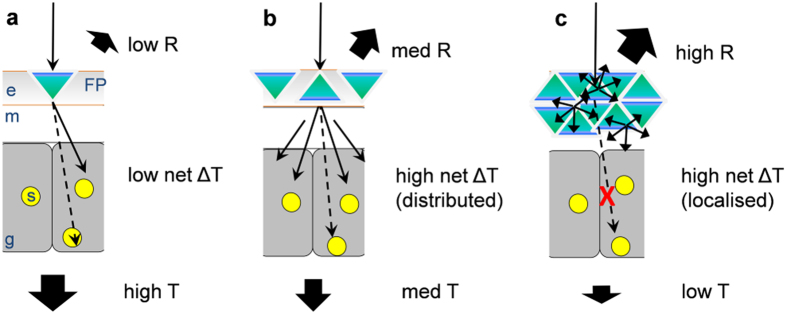
Hypothetical mechanism for non-linearity of tissue heating in coral tissue with FPs. Three cases of FP density are shown, (**a**) low FP, (**b**) medium FP and (**c**) high FP. The vertical coral tissue structure shows the oral tissue layer with the epidermis (**e**) the connecting tissue layer (mesoglea; m), gastrodermal host cells (**g**) containing *Symbiodinium* photosymbionts (yellow circles; s). The scheme ignores the lower tissue layer (aboral tissue) and the skeleton (see text) and assumes that (i) fluorescent pigments (blue-green triangles; FP) are situated above the symbionts, (ii) that skeleton scattering is low, and (iii) that tissue background scattering is constant for (**a**–**c**). Incident light (black arrow) is either reflected (R), absorbed and subsequently dissipated as heat (ΔT), or transmitted and lost from the system (T). (**a**) Light reaches FPs, causing scattering and enhanced chance of symbiont photon absorption and heating. Part of the light reaches lower layers (dotted black arrow) where symbionts absorb light and dissipate heat. For low FP content the scattering effect is relatively small and transmission through the coral is high. (**b**) As FP content increases, light scattering is enhanced, causing multiple scattering between FP granules. This strongly enhances net heating of the coral tissue system. Penetration depth and transmission is somewhat reduced. Reflectivity is enhanced because of diffuse backscattering by FP. (**c**) At high densities of FP, strong diffuse scattering enhances reflectivity and heating by symbionts located in proximity to the FPs. However, strong scattering follows reduced penetration depth and thus reduced chance of photon absorption and heating for deeper tissue layers (red cross on dotted black arrow). Net heating is high but caused by localized scattering of a small part within the tissue. As light scattering increases further, heating will eventually decrease and most of the light is lost as diffuse reflectance. The model was supported by Monte Carlo simulations of photon transport (Fig. S4).

However, our results also showed that heating saturated at high levels of green fluorescence, suggesting that the effect of FPs on coral light absorption and heating become non-linear at high irradiance (Fig. 7). In order to illustrate the potential mechanism underlying such non-linearity, we developed a conceptual model (Fig. 8) based on basic light scattering theory and a simplified Monte Carlo simulation of photon propagation (Fig. S4). The model is further described in the supplementary information. The model assumes three simplified tissue layers, a top layer that contains the FP scattering elements^51^ at varying densities (representative of the epidermal coral tissue layer), a middle layer of high absorption (representative of the gastrodermal layer containing *Symbiodinium* and/or other light absorbing elements such as chromoproteins), and a bottom layer of low absorption and low scattering (representative of the coral skeleton^12^). When light scattering in the top layer is low (low FP content) most of the incident radiation penetrates through the coral and thus light absorption and heating is low (Fig. 8a). At intermediate densities of light scattering (intermediate FP content), the incident irradiance is strongly diffused by the FP layer, which leads to an enhanced chance of photon absorption and heating in the lower layers (Fig. 8b). Because light scattering also increases the diffuse radiant flux that escapes the tissue (Fig. S4), both diffuse reflectance and absorption/heating is increased relative to the low FP scenario. However, as light scattering in the top layer further increases, absorption/heating will eventually saturate. This saturation is because intense scattering will reduces light penetration into coral tissue and thus leads to reduced chance of light absorption in deeper light absorbing layers below (Figs 8c and S4d). Thus we suggest that the observed heating saturation at higher irradiance is caused by reduced light penetration because of intense scattering by FPs in the upper tissue layers. This theory implies that at excess densities of light scattering FPs, heating will decrease because more of the light is scattered away and lost from the system as diffuse reflectance (Fig. S4d).

We found that coral tissues with highest green fluorescence revealed a high reflectivity of approximately 20% (Fig. 2e), in line with previously measured reflectivity of fluorescent corals reaching up to 15%^18^. It has been suggested that the high reflectivity of FPs would lead to reduced tissue light absorption and tissue heating^15^. The results of this study however suggest that such a photoprotective function is likely to be FP density dependent, where low densities of light scattering FPs lead to an increase of both reflectivity and light absorption/heating (Figs 8 and S4a,b), while high densities will lead to increased tissue reflectivity and eventually reduce light absorption under high irradiance (Fig. S4d). With regard to the ongoing debate concerning the photoprotective or photosynthesis-stimulating function of FPs in corals, future studies need to consider the potential for density dependent effects of FPs on coral photobiology.

The thermal action spectrum showed lowest heating for blue light excitation when normalized to equal power for each spectral band (500 W m^−2^, Fig. 5a–d). In contrast, previous studies performed with brown non-fluorescent corals (*Porites lobata* and *Stylophora pistillata*) found that heating was greatest when excited with blue light. It was shown that heating was dominated by the absorption spectrum of *Symbiodinium* with peak absorption of Chl *a* in the blue (at 430 nm^15^). Assuming that the Stokes shift from blue to green would occur before light reaches *Symbiodinium*, as would be the case for endodermally located FPs^51^, *Symbiodinium* would be exposed to reduced blue and enriched green light. Green light absorption in *Symbiodinium* is due to peridinin and the PCP complex^54^, of which the total absorption cross-section is lower than for Chl *a* in the blue^46^. Thus the comparably lower heating for the blue spectral band (Fig. 5a–d) could be caused by significant re-emission of green fluorescence following blue light absorption^19^ (Fig. 6).

Heating peaked when excited with the red spectral band peaking at around 680 nm (Fig. 5). This red spectral band overlaps with the second major absorption peak of *Symbiodinium* chlorophyll *a* at 675 nm^55^, thus suggesting a major contribution of Chl *a* absorption to the heating of coral tissue. Additionally, it is possible that non-fluorescent chromoproteins with high molecular extinction coefficients but low fluorescence (e.g. purple-blue chromoproteins) could have contributed to the characteristic heating patterns observed here^19^. Hyperspectral imaging suggested the presence of cyan and green FPs for HF fragments and only green FPs for NF fragments (Fig. 2e), which matches the previous observation that especially cyan FPs are light inducible^39^. The wavelength-dependent heating in fluorescent corals is likely a function of the specific mixture of different GFP-like proteins present. A detailed molecular analysis of the type and quantity of GFP-like proteins was beyond the scope of the present study, which focussed on linking coral optics to microscale heating in relation to green fluorescence. Future studies performing such detailed microscale measurements aligned with FP analysis of small tissue sections will provide an important next step in understanding the radiative energy regulation in green fluorescent corals.

It is important to consider other factors that could affect the absolute heating measured in the investigated tissues. Enhanced skeleton scattering within the corallite could contribute to the enhanced polyp tissue scalar irradiance, which would thus affect tissue heating^13^,^17^. However, in thick-tissued corals, such an effect is likely to be negligible^56^. We did not quantify zooxanthellae density as our microscale measurement approach would require determining the cell density for microscopic tissue sections. Thus it is possible that tissues could have different zooxanthellae densities which would affect tissue heating. Likewise, differences in the thickness of the thermal boundary layer (TBL) between coenosarc and polyp tissues can affect the measured surface heating^34^. However, a potential TBL effect cannot explain the enhanced heating of polyp areas for the HF vs. the NF fragment (Fig. 7b). Although we cannot account for such factors, the positive correlation between green fluorescence and tissue heating independent of light acclimation and coral topography or tissue type (Fig. 4), strongly suggests that the enhanced heating is caused by simple light scattering mechanisms underlying the presence of fluorescent pigments^18^.

Quenching analysis performed with PAM showed no difference in the effective quantum yield of PSII between tissues with different green fluorescence levels (Fig. 3b). Previous studies aimed at investigating the effects of FPs on the quantum yield of PSII have produced inconclusive results. For instance, Salih *et al*.^18^ found enhanced maximum quantum yields of PSII in green fluorescent morphs of *Acorpora palifera*, as compared to their non-fluorescent counterparts. Likewise, Roth *et al*.^41^ studied the effects of temperature stress on corals with FPs and showed that there was a positive correlation between green fluorescence and the effective quantum yield of PSII in healthy corals of *Acropora yongeii*. However, other studies did not find significant effects of GFP-like proteins on variable Chl *a* fluorometry^57^,^58^. For instance, no difference was found in the maximum quantum yield of PSII between fluorescent and non-fluorescent morphs of the coral *Galaxea fascicularis*^57^. Likewise, no difference was found in any photosynthetic parameter for different fluorescent morphs of *Leptoseris spp*.^58^.

While our results did not reveal any difference in the quantum yield of PSII (Fig. 3b), they did show minor but significant differences in Y(NPQ) and Y(NO) in tissues with different levels of green fluorescence. HF polyp tissues showed lower NPQ under excess irradiance compared to HF coenosarc tissues with lower FP content (Fig. 3d). Since NPQ is a photoprotective mechanism that dissipates excess energy^59^ and usually increases at the expense of the photosynthetic quantum yield, we would have expected a reduction in the effective quantum yield between these tissue types. However, we found no difference in the photosynthetic quantum yield but an increase in the non-regulated energy pathway, Y(NO) for HF polyp tissues (Fig. 3e). This suggests that tissues with high amounts of FPs might be able to get rid of excess energy by other means not accounted for by NPQ. Such excess energy dissipation could be related to the photoprotective properties of FP^18^,^60^.

In summary, the present study investigated potential links between green fluorescence and heating of coral tissues. We showed that both coral tissue heating and scalar irradiance increased with green fluorescence and there was a positive correlation between the enhancement of tissue scalar irradiance and coral heating, albeit heating saturated at maximal levels of measured green fluorescence. Our results suggest that FPs scatter and diffuse the incident radiation, which subsequently enhances light absorption and heating of the coral tissue. However, heating saturates as strong light scattering reduces the vertical penetration depth through the coral tissue eventually leading to a reduction in light absorption at high levels of fluorescent pigments. We conclude that fluorescent pigments in corals can have a central role in modulating light absorption and heating of corals both via their fluorescent and light scattering properties.

## Additional Information

**How to cite this article**: Lyndby, N. H. *et al*. Heat generation and light scattering of green fluorescent protein-like pigments in coral tissue. *Sci. Rep*. **6**, 26599; doi: 10.1038/srep26599 (2016).

## Supplementary Material

Supplementary Information

## Figures and Tables

**Table 1:** Average green fluorescence (500–585 nm) emission of *Dipsastraea* sp.

Tissue area	HF	NF
Polyp	0.90 ± 0.051	0.09 ± 0.017
Coenosarc	0.05 ± 0.002	0.02 ± 0.003

Green fluorescence (a.u., means ± s.e.m) was imaged on three polyp and coenosarc tissues for each fragment (HF – highly fluorescent and NF – normal fluorescent, n = 3). Data were normalized to maximal values of measured fluorescence.
