# Supplementary material for: Heat generation and light scattering of green fluorescent protein-like pigments in coral tissue
Source: Sci Rep. 2016 May 26;6:26599. doi: 10.1038/srep26599 (PMC4880895; doi:10.1038/srep26599)
Supplement: Supplementary Information [file srep26599-s1.pdf]

Supplementary Information:

Heat generation and light scattering of green fluorescent protein-like pigments in coral tissue

Running title:

Coral heating and FPs

Niclas H. Lyndby[a], Michael Kühl[a,b,1], and Daniel Wangpraseurt[a,b,1]

[a]Marine Biological Section, Department of Biology, University of Copenhagen, DK-3000 Helsingør, Denmark

[b]Plant Functional Biology and Climate Change Cluster, University of Technology Sydney, New South Wales 2007, Australia

[1]Corresponding authors: Daniel.wangpraseurt@bio.ku.dk, mkuhl@bio.ku.dk

Supplementary Figures

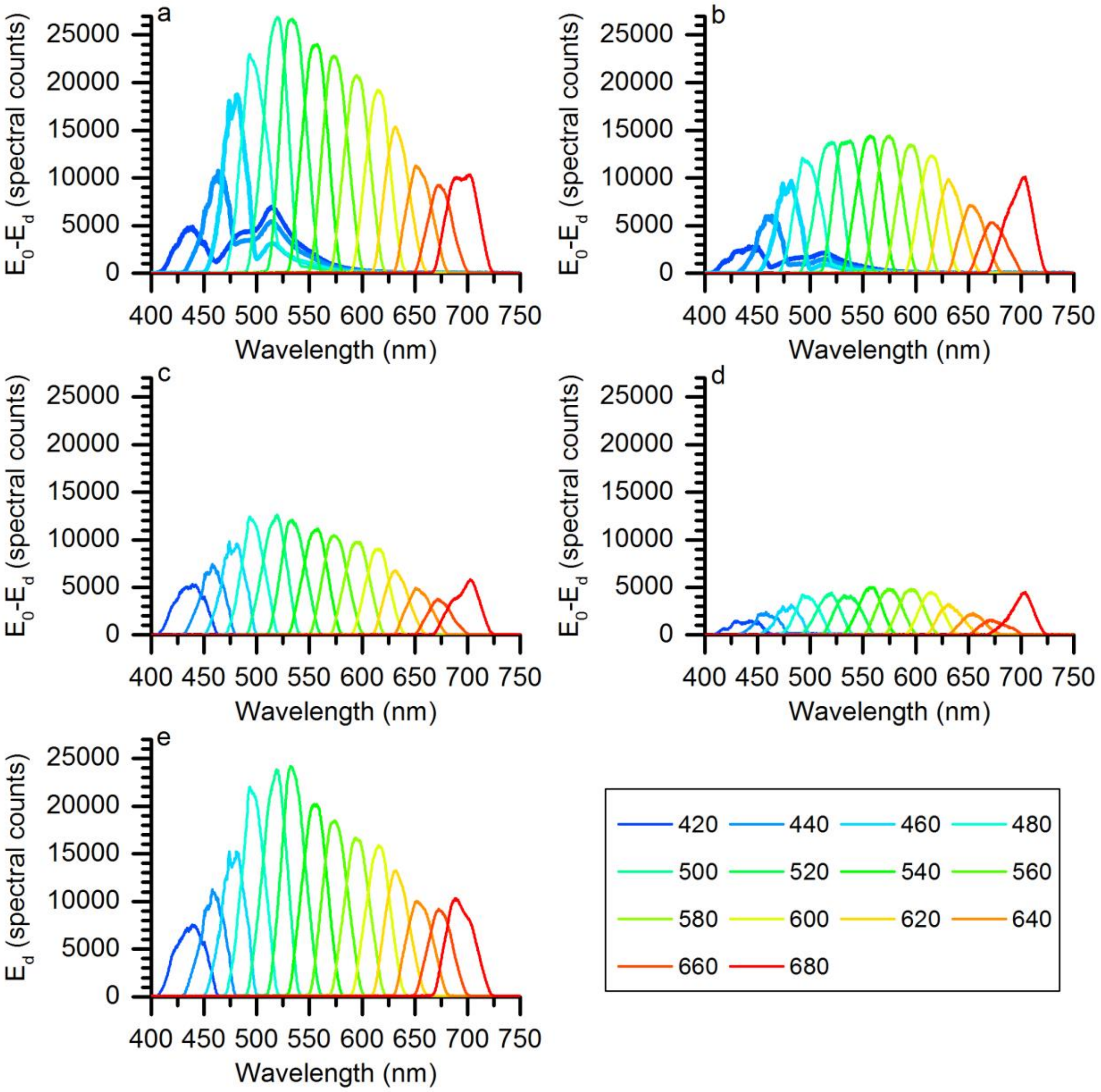

Figure S1: Coral tissue surface scalar irradiance as a function of wavelength. The figure is complimentary to Figure 6. (a-d) Enhancement of spectral scalar irradiance (coral tissue surface E_0 - E_d) at the tissue surface of the a) HF polyp, b) NF polyp, c) HF coenosarc and d) NF coenosarc tissues (*n*=3). (e) Spectral distribution of the incident downwelling irradiance for the different wavebands used for illumination. The incident spectral irradiance was delivered in steps of 20 nm between 400-700 nm

(see methods). Thick lines in (a) and (b) represent spectral bands for which green fluorescence was pronounced. For clarity only means are shown; n=3.

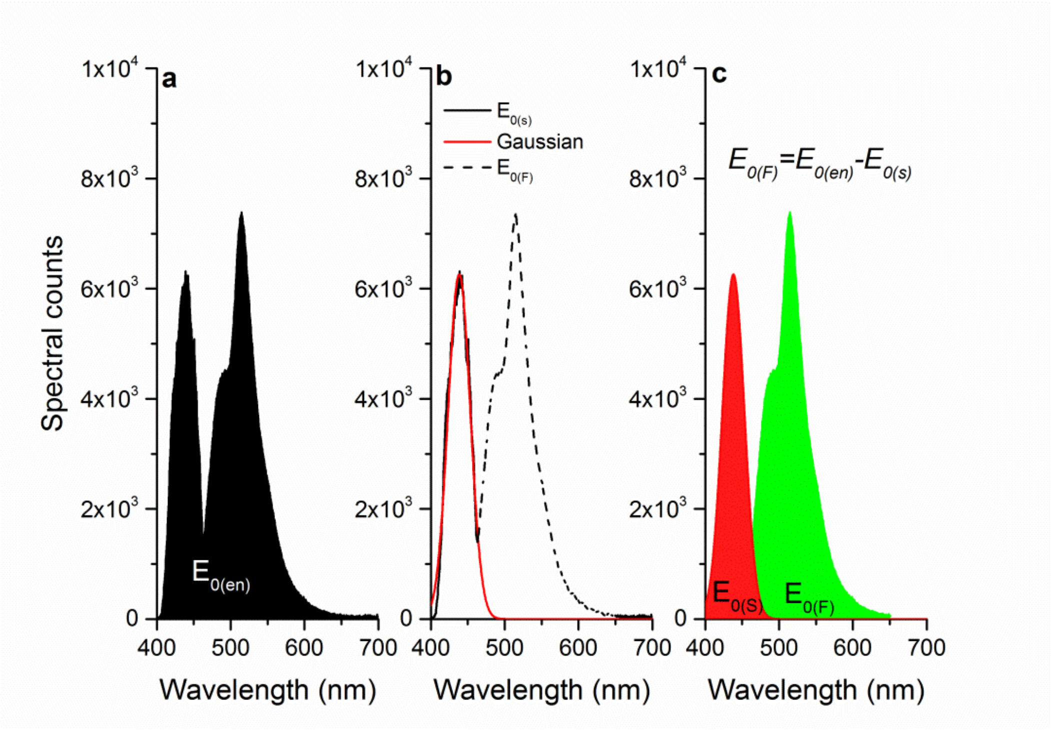

Fig. S2. Separation of scattering and fluorescence contributions to E_0 enhancement. (a) The total enhancement of scalar irradiance was $E_{0(en)} = [\int_{400}^{700} E_0(\lambda)d\lambda] - [\int_{400}^{700} E_d(\lambda)d\lambda]$ b) The fluorescence contribution to $E_{0(en)}$ was approximated based on the Stokes shifted spectrum (dashed black line) relative to the spectral distribution of the incident light spectrum. Data points indicative of fluorescence between 420-620 nm were removed (dashed black line) and the spectrum (black line) was fitted to a Gaussian distribution (red line) based on a non-linear least squares Levenberg–Marquardt iteration algorithm (R^2>0.98). c) The contribution of scattering (red area) was $E_{0(S)} = \int_{400}^{700} Gaus(\lambda)d\lambda$ and the contribution of fluorescence was $E_{0(F)} = E_{0(en)} - E_{0(S)}$ and was finally expressed as percentage of the enhancement factor (see main text).

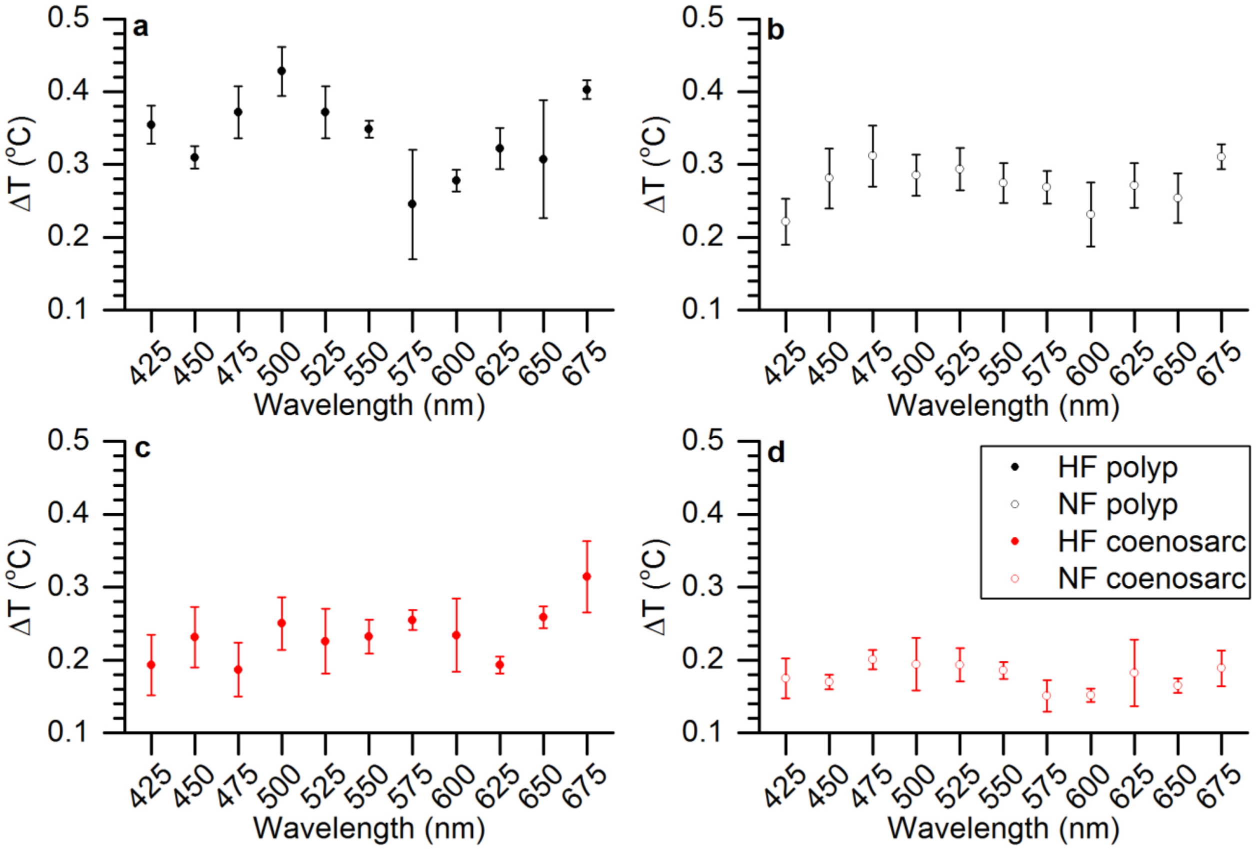

Figure S3: Coral thermal action spectrum normalized for equal photon irradiance. Coral tissue surface heating is expressed as ΔT, i. e., the difference between coral surface temperature and the temperature in the ambient water, and was normalized for to a photon irradiance of 418 µmol photons m^{-2} s^{-1} (±1 µmol photons m^{-2} s^{-1}) over each spectral band. Measurements were performed for a) HF polyp, b) NF polyp, c) HF coenosarc and d) NF coenosarc tissues (*n*=3).

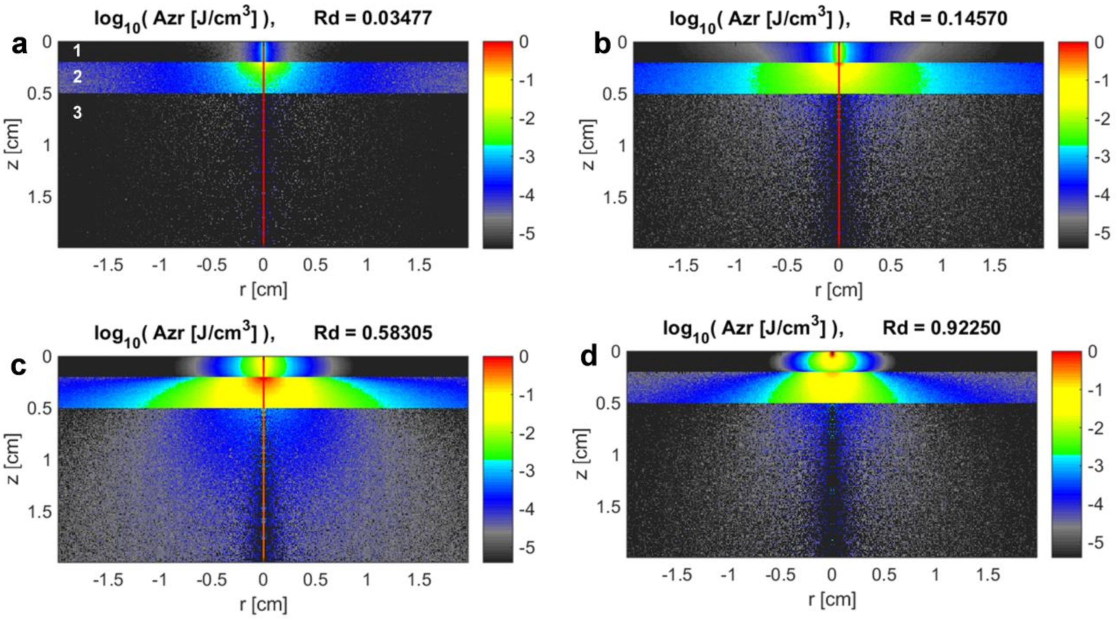

Figure S4. Monte Carlo simulations of light absorption in a simplified multilayered coral structure. The simulation was developed to illustrate and support basic principles of light scattering and absorption/heating in multilayered biological tissues. Monte Carlo simulations are stochastic models that are used in medical tissue optics to simulate photon propagation and score physical quantities (diffuse reflectance, absorbance, transmittance) based on a set of optical properties[1]. The present work used the multilayered Monte Carlo model (MCML[2]) which is regarded as the 'gold standard' in modeling photon transport in turbid media.

The model uses 3 layers (1, 2, 3) noted with white font in (a). The top layer (1) is the fluorescent pigment layer (representative of the epidermal coral tissue layer), the middle layer (2) is the light absorbing layer (representative of the photosymbiont-containing gastrodermal layer), the bottom layer (3) is a light distributing scattering layer (representative of the coral skeleton). The actual number of tissue layers of the entire coral tissue is higher but including further complexity is not needed in this model, as it aims at illustrating how changes in light scattering of a top layer can affect light absorption in layers below.

The optical properties of layer 2 and 3 were fixed in all cases (a-d) and only the optical properties of the fluorescent pigment layer were changed. The model assumes isotropic scattering throughout, i. e., the anisotropy of scattering g = 0. The optical properties of layer 3 were chosen to be mainly light

distributing and characterized by low absorption and low scattering. The properties were set to: absorption coefficient (μ_a) = 0.001 cm^{-1}, scattering coefficient (μ_s) = 0.001 cm^{-1}.

The optical properties of layer 2 were chosen to be light absorbing, representative of the gastrodermal layer (or a combination of tissue layers where light is absorbed either by chromoproteins and/or *Symbiodinium* cells). The optical properties of layer 2 were set to: μ_a = 0.1 cm^{-1}, μ_s = 0.001 cm^{-1}.

The optical properties of layer 1 were chosen to be the light scattering layer, characterized by varying densities of light scattering fluorescent host pigments. The scattering was for a) μ_s = 0.1 cm^{-1}, b) μ_s = 1 cm^{-1}, c) μ_s = 10 cm^{-1}, and d) μ_s = 100 cm^{-1}. The absorption coefficient was constant (μ_a = 0.001 cm^{-1}).

The model scores light absorption, which is shown as $\log_{10}$ (Azr [in J/cm^3]) in a false colour scale. The model was developed in 2-D, where depth (z) is modelled from the tissue surface down to 1.8 cm. The model extends uniformly along the y-axis (r). An infinitely narrow photon beam is delivered as vertically incident irradiance at the centre of the model (red line). The diffuse reflectance (R_d), i.e. the photon flux that escapes the tissue after specular reflection and/or multiple scattering is shown as a fraction of 1 (where 1= total reflectivity).

The model illustrates that differences in the scattering of a single top layer (1) can lead to a non-linear behavior of heating/absorption ($\log_{10}$ (Azr [in J/cm^3]). Low scattering (a) of the top layer leads to low heating and low diffuse reflectance, while higher scattering (b-c) increases both diffuse reflectance and heating. At very high densities (d) of the top light scattering layer, heating is reduced because of reduced vertical penetration depth and most of the light escapes as diffuse reflectance.

Supporting References

1. Tuchin VV. *Tissue optics: light scattering methods and instruments for medical diagnosis*. SPIE press Bellingham (2007).

2. Wang L, Jacques SL, Zheng L. MCML—Monte Carlo modeling of light transport in multi-layered tissues. *Computer Methods and Programs in Biomedicine* **47**, 131-146 (1995).
